# Cortical iron mediates age‐related decline in fluid cognition

**DOI:** 10.1002/hbm.25706

**Published:** 2021-12-02

**Authors:** Cortney M. Howard, Shivangi Jain, Angela D. Cook, Lauren E. Packard, Hollie A. Mullin, Nan‐kuei Chen, Chunlei Liu, Allen W. Song, David J. Madden

**Affiliations:** ^1^ Center for Cognitive Neuroscience Duke University Durham North Carolina USA; ^2^ Brain Imaging and Analysis Center Duke University Medical Center Durham North Carolina USA; ^3^ Department of Psychiatry and Behavioral Sciences Duke University Medical Center Durham North Carolina USA; ^4^ Present address: Department of Psychological and Brain Sciences University of Iowa Iowa City Iowa USA; ^5^ Present address: Department of Biomedical Engineering University of Arizona Tucson Arizona USA; ^6^ Present address: Department of Electrical Engineering and Computer Sciences University of California Berkeley California USA

**Keywords:** aging, biomarkers, brain iron, cognition, susceptibility

## Abstract

Brain iron dyshomeostasis disrupts various critical cellular functions, and age‐related iron accumulation may contribute to deficient neurotransmission and cell death. While recent studies have linked excessive brain iron to cognitive function in the context of neurodegenerative disease, little is known regarding the role of brain iron accumulation in cognitive aging in healthy adults. Further, previous studies have focused primarily on deep gray matter regions, where the level of iron deposition is highest. However, recent evidence suggests that cortical iron may also contribute to cognitive deficit and neurodegenerative disease. Here, we used quantitative susceptibility mapping (QSM) to measure brain iron in 67 healthy participants 18–78 years of age. Speed‐dependent (fluid) cognition was assessed from a battery of 12 psychometric and computer‐based tests. From voxelwise QSM analyses, we found that QSM susceptibility values were negatively associated with fluid cognition in the right inferior temporal gyrus, bilateral putamen, posterior cingulate gyrus, motor, and premotor cortices. Mediation analysis indicated that susceptibility in the right inferior temporal gyrus was a significant mediator of the relation between age and fluid cognition, and similar effects were evident for the left inferior temporal gyrus at a lower statistical threshold. Additionally, age and right inferior temporal gyrus susceptibility interacted to predict fluid cognition, such that brain iron was negatively associated with a cognitive decline for adults over 45 years of age. These findings suggest that iron may have a mediating role in cognitive decline and may be an early biomarker of neurodegenerative disease.

## Graphical abstract

This study utilized a voxelwise, quantitative susceptibility mapping (QSM) approach and demonstrates that QSM is a valid brain iron measure. When coupled with voxelwise methods, QSM can elucidate iron‐age and iron‐cognition relations. Brain iron in the inferior temporal cortex mediates the association between age and fluid cognition. Additionally, age and right inferior temporal cortex susceptibility interact to predict fluid cognition such that brain iron is negatively associated with cognitive decline for adults over 45 years. This novel finding suggests that iron has a causal role in cognitive decline, is a critical source of variance in cognitive aging studies, and may be a biomarker of neurodegenerative disease.

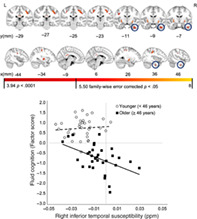

## 1 INTRODUCTION

Iron is the most abundant metal in the brain and is critical for several biological mechanisms that support neuronal functioning, including oxygen transportation, myelination, neurotransmitter synthesis, and mitochondrial respiration (Gutteridge, 1992; Koeppen, 1995; Rouault & Cooperman, 2006; Todorich, Pasquini, Garcia, Paez, & Connor, 2009). Brain iron concentration is not distributed uniformly, and deep gray matter regions exhibit the highest iron levels, although iron is detectable in cortical regions (Haacke et al., 2005; Morris, Candy, Oakley, Bloxham, & Edwardson, 1992). The association of increased deep gray matter iron with the neuropathology of Parkinson's diseases is well known (Gotz, Double, Gerlach, Youdim, & Riederer, 2004; He et al., 2015; Sofic, Paulus, Jellinger, Riederer, & Youdim, 1991). Recent evidence suggests that increased accumulation of brain iron may have a role in Alzheimer's disease as well, by interacting with amyloid‐related neurodegeneration and increasing oxidative stress (Ayton, Lei, & Bush, 2013; Bush, 2013; Cornett, Markesbery, & Ehmann, 1998; Schubert & Chevion, 1995; Tao, Wang, Rogers, & Wang, 2014). Additionally, there is evidence that iron chelators reduce amyloid plaques and memory impairment in Alzheimer mouse models (Cherny et al., 2001; Grossi et al., 2009) and in humans (Ibach, Haen, Marienhagen, & Hajak, 2005; Ritchie et al., 2003). Researchers have begun utilizing MRI techniques to measure brain iron in vivo, including R2* relaxometry and quantitative susceptibility mapping (QSM). While both techniques are validated measures of brain iron (Langkammer et al., 2012; Sun et al., 2015; Zheng, Nichol, Liu, Cheng, & Haacke, 2013), QSM is more iron selective, reproducible, and less dependent on field strength than relaxometry (Ghassaban, Liu, Jiang, & Haacke, 2019; Li et al., 2015; Liu, Li, Tong, Yeom, & Kuzminski, 2015).

In human development, iron accumulates in basal ganglia and deep gray matter regions through adolescence (Li et al., 2014). Some regions, such as the putamen, continue to accumulate iron throughout later adulthood (Bartzokis et al., 1997; Hallgren & Sourander, 1958; Ramos et al., 2014). While the level of iron is lower in the cortex than in the deep gray matter, age‐related iron accumulation is detectable throughout the cerebral cortex (Khattar et al., 2021). The significance of age‐related increases in brain iron is not yet entirely understood. However, neurobiological evidence suggests that iron deposition, beyond some (unknown) threshold, contributes to dysregulation of neuronal mechanisms that may disrupt neurotransmission and increase neurotoxicity, even in the absence of frank disease (Becerril‐Ortega, Bordji, Fréret, Rush, & Buisson, 2014; Gutteridge, 1992; Hare, Ayton, Bush, & Lei, 2013; Li & Reichmann, 2016; Zecca, Youdim, Riederer, Connor, & Crichton, 2004). However, it is unknown if the iron‐related neuronal disruptions are attributable to failures of the iron regulatory systems or dysfunctional iron‐related proteins (e.g., ferritin). Beyond the role of iron in neuropathology, evidence from both QSM and histology suggests that region‐specific iron may be a biomarker for behavioral and cognitive decline in Parkinson's disease (Uchida et al., 2019) and Alzheimer's disease (Ayton et al., 2017; Ayton et al., 2019; Sun et al., 2017). The accumulation of brain iron in healthy aging has been linked to deficits in working memory, episodic memory, and motor performance (Bartzokis, Tishler, Shin, Lu, & Cummings, 2004; Daugherty, Haacke, & Raz, 2015; Li et al., 2015; Penke et al., 2012; Raz & Daugherty, 2018; Sullivan, Adalsteinsson, Rohlfing, & Pfefferbaum, 2009; Van Bergen et al., 2018; Zachariou et al., 2020).

Most studies investigating brain iron in aging and neurodegenerative diseases have analyzed relations within predefined regions of interest (ROIs), particularly those in deep gray matter. This approach is not sensitive to effects beyond these ROIs and involves estimating iron across large cortical surfaces. Thus, whole‐brain investigations may reveal novel findings. For example, whole‐brain QSM analyses of Alzheimer's disease (Acosta‐Cabronero et al., 2013) and Parkinson's disease (Uchida et al., 2019) suggest that iron deposition in distinct cortical regions contributes to neuropathology. Additionally, in healthy aging, Acosta‐Cabronero, Betts, Cardenas‐Blanco, Yang, and Nestor (2016) reported age‐iron relations in basal ganglia and frontal motor regions by utilizing a whole‐brain approach. Investigation of iron in cortical as well as deep gray matter regions may consequently help to clarify the neural mechanisms of age‐related decline in cognition, which is most pronounced for speed‐dependent (fluid) and sensorimotor functioning (Baltes & Lindenberger, 1997; Craik & Salthouse, 2000; Salthouse, 1992, 1996; Salthouse & Madden, 2007). Zachariou et al. (2020), for example, found that within a group of older adults, high cortical iron concentration was associated with disrupted functional connectivity of frontoparietal networks and reduced working memory performance. To our knowledge, however, there has been no whole‐brain or voxelwise analysis investigating the relation of brain iron to age‐related differences in cognitive function.

Here, we used a whole‐brain, voxelwise QSM analysis to investigate age‐related differences in relation between brain iron and fluid cognition, in healthy adults. We expected to replicate previous studies demonstrating age‐related brain iron accumulation within frontal sensorimotor regions (Acosta‐Cabronero et al., 2016; Zachariou et al., 2020). Additionally, we hypothesized that cortical brain iron, particularly within visual and sensorimotor regions, mediates age‐related decline in fluid cognition, in view of the strong dependence of age‐related cognitive decline on sensory functioning (Baltes & Lindenberger, 1997; Monge & Madden, 2016; Schneider & Pichora‐Fuller, 2000). Further, we hypothesized that brain iron is a relatively early biomarker of age‐related declines in fluid cognition, such that the brain iron‐cognition association is stronger in mid to late life. We first validated our QSM measures with data from histology and then utilized voxelwise QSM to analyze the relation of iron to both age and fluid cognition. We then tested whether brain iron within identified cognition‐related clusters has a mediating role in the age‐cognition relation. Finally, we investigated differences in the iron‐cognition relation across age.

## 2 METHODS

### 2.1 Participants

Ninety‐eight community‐dwelling individuals between 18 and 78 years of age gave written informed consent and were enrolled in the study protocol approved by the Duke University Institutional Review Board. All participants were compensated for their time and completed an initial screening (Session 1) that included three psychometric tests: the vocabulary and logical memory subtests of the Wechsler Adult Intelligence Scale‐Revised (WAIS‐R; Wechsler, 1981), the Mini‐Mental State Exam (MMSE; Folstein, Folstein, & Mchugh, 1975), and the Beck Depression Inventory (BDI; Beck, 1978). Participants were excluded if the scaled score on the WAIS vocabulary subtest was less than 10 (50th percentile), the MMSE score was less than 27, or the BDI score was greater than 16. See Table 1 for participant demographics and inclusion measures. During Session 1, participants also performed 12 tests of fluid cognition, detailed below. Imaging was conducted approximately 1 month later in Session 2. Eleven participants were excluded based on Session 1 screening criteria. Six individuals declined to participate in the subsequent MRI session, and five additional participants failed MRI safety screening. Finally, nine participants were excluded for poor image quality (due to either motion or missing slices due to technical errors). The final sample consisted of 67 participants (35 females), with 26 participants between the ages of 18 and 39 years (*M* = 28 years, *SD* = 5.36), 20 participants between the ages of 40 and 59 years (*M* = 49 years, *SD* = 5.74), and 21 participants between the ages of 60 and 78 years (*M* = 70 years, *SD* = 4.87).

**TABLE 1:** Participant characteristics

	Range	Mean	Standard deviation	*r* with age
Age	18–78	46.22	17.99	—
Education	12–20	17.41	2.29	.006
WAIS vocabulary	11–19	15.04	2.03	−.197
MMSE	28–30	29.59	0.60	.004
BDI	0–15	2.65	3.20	.077

*Note*: *N* = 67 (35 female).

Abbreviations: BDI, Beck Depression Inventory; MMSE, Mini‐Mental State Exam; WAIS Vocabulary, Wechsler Adult Intelligence Scale‐Revised Vocabulary scaled score.

### 2.2 Cognitive assessments

Cognitive testing assessed three domains of fluid cognition: perceptual‐motor speed, executive function, and episodic memory. Twelve neurocognitive tests were administered, comprising four tests for each domain. These were a combination of computer‐based tests of reaction time (RT) and standardized psychometric tests. Computer‐administered tests were developed in‐house and described in more detail in Madden et al. (2017a). Each domain included one test from the cognition section of the NIH Toolbox (Gershon et al., 2013).

Perceptual‐motor speed was measured from three computer‐administered RT tests (simple RT and two versions of choice RT) and number correct in 85 s from the NIH Toolbox Pattern Comparison Test. Executive function comprised two computer‐administered tests (two‐choice digit/symbol comparison RT and flanker task incompatible RT divided by compatible RT), a standardized psychometric test (Trails B minus Trails A; Reitan, 1986), and the computed score on the NIH Toolbox Dimensional Change Card Sort Test. We assessed episodic memory from two computer‐administered tests (a 6‐item shape change detection task; Saults & Cowan, 2007; and 20‐min delayed recall of 16 words), one psychometric test (WAIS logical memory delayed; Wechsler, 1997), and the computed score for the NIH Toolbox Picture Sequence Memory Test.

We used a factor analytic approach (Dagley et al., 2017; Hedden et al., 2012, 2014; Madden et al., 2017b; Salthouse et al., 2015) to define a latent construct for perceptual speed, executive function, and memory in terms of the first factor for the four tests in each of these domains. We extracted the first unrotated factor from a factor analysis of the four tests in each domain. We conducted a principal axis factor analysis on the relevant indicator variables (for all participants) and used the factor score (for each participant) from the first unrotated factor as the summary measure. We used a principal factor analysis rather than a principal components analysis because our interest was in the shared variance among the indicator variables rather than the mathematically independent components (Salthouse et al., 2015). The factor scores served as summary measures for data reduction. An overall measure of fluid cognition was derived from the first factor derived from all 12 tests and the factor scores were covaried for sex and WAIS vocabulary.

### 2.3 Image acquisition

Anatomical imaging data were collected on a 3T GE Signa Ultra High Performance (UHP) Signa MR360 whole‐body 60 cm bore MRI scanner (GE Healthcare, Waukesha, WI) equipped with a peak strength of 113 mT/m gradients and a 260 T/m/s slew rate. The scanner possessed a 48‐channel head coil that was used for radiofrequency reception. Participants wore earplugs to reduce scanner noise, and foam pads were used to minimize head motion. Three‐plane (straight axial/coronal/sagittal) localizer fast spin‐echo images were acquired at the start of the scan to define the data collection volume. Global field homogeneity was ensured by the use of a semi‐automated high‐order shimming program. One run of T1‐weighted anatomical images, one resting‐state run of T2*‐weighted (functional) BOLD (blood‐oxygen‐level‐dependent) contrast imaging, one run of Susceptibility Weighted Angiography (SWAN) sequence, four runs of event‐related T2*‐weighted imaging, one run of DWI, and one run of T2‐weighted FLAIR imaging were recorded. The DWI, task, and resting‐state imaging data are not reported here.

Anatomical T1‐weighted images included 292 straight axial slices that were attained using a 3D fast inverse‐recovery‐prepared spoiled gradient recalled (SPGR) sequence with repetition time (TR) = 2,203.5 ms, echo time (TE) = 3.076 ms, inversion recovery time (TI) = 900 ms, field of view (FOV) = 240 mm × 240 mm, flip angle = 8°, voxel size = 1 × 1 × 1 mm, acquisition matrix size = 240 × 240 mm, and a sensitivity encoding (SENSE) factor of 2, using the array spatial sensitivity encoding technique and extended dynamic range.

SWAN images were obtained while participants practiced an fMRI task with 8 echoes to obtain magnitude and phase imaging under the following parameters: mean TE = 24.844 ms, TR = 40.5 ms, flip angle = 15°, FOV = 256 mm × 256 mm, acquisition matrix size = 256 × 256 mm, voxel size = 1 × 1 × 1 mm, number of slices = 148 and a SENSE factor of 2.

### 2.4 Susceptibility estimation

Susceptibility map reconstruction was performed with STI Suite v 3.0 (https://people.eecs.berkeley.edu/~chunlei.liu/software.html) in MATLAB (version 2017a) and included phase unwrapping, background field removal (necessary because of the air‐tissue interface), and susceptibility reconstruction. The STI Suite utilized the 3D Axial SWAN scans, and a binary brain mask was extracted from the average magnitude image using functional magnetic resonance imaging of the brain (FMRIB) software brain extraction tool (Jenkinson, Beckmann, Behrens, Woolrich, & Smith, 2012). Using the unwrapped phase image and the binary brain mask, the improved sparse linear equation and least‐squares (iLSQR) (Li, Wu, & Liu, 2011) algorithm was used to reconstruct the susceptibility map.

### 2.5 Validation of susceptibility data

To validate the voxelwise susceptibility analyses, we compared the susceptibility data to iron concentrations, in deep gray matter ROIs, derived from postmortem histology (Hallgren & Sourander, 1958). To obtain these ROI data, individual T1 images for each participant were registered to the 3D Axial SWAN images using FLIRT (FMRIB's Linear Image Registration Tool) set to 6° of freedom. The T1 image was then linearly registered to Montreal Neurological Institute (MNI) space using FLIRT to create an affine matrix and nonlinearly registered to MNI space using FNIRT (FMRIB's Nonlinear Image Registration Tool). The nonlinear transformation was then inverse transformed from MNI to native space. Atlas regions for nine subcortical ROIs, including the substantia nigra, red nucleus, putamen, thalamus, globus pallidus, caudate nucleus, amygdala, hippocampus, and dentate nucleus, were obtained from a developmental atlas devised for QSM data (Zhang et al., 2018). The *Fslstats* tool was then used to calculate the mean susceptibility of each ROI. The ROIs were averaged across the left and right hemispheres. The substantia nigra subdivisions and the thalamus subdivisions were combined, and left/right averaged to obtain a single value for the substantia nigra and a single value for the thalamus.

The comparison data were iron concentrations derived from postmortem histology (Hallgren & Sourander, 1958). Using histological analysis of 81 patient brains (ages 30–100 years) and 17 brains of young people (ages 0–29 years), Hallgren and Sourander documented higher iron deposition in subcortical regions relative to cortical regions and graphed the average iron levels in several regions. During adulthood, pronounced increases in iron content with increasing age were evident in the putamen and caudate nucleus. We extracted comparison data from the Hallgren and Sourander tables and used the DataThief program (https://datathief.org/) to estimate the raw data presented only in figures. We selected comparison data from the Hallgren and Sourander data set for individuals 18–79 years of age. The resulting sample size was 55 participants for globus pallidus (*M* age = 47.41 years, *SD* = 17.62), 54 participants for putamen (*M* age = 46.14 years, *SD* = 17.26) and 52 participants for thalamus (*M* age = 47.42 years, *SD* = 18.44). We averaged the iron concentrations across patients in these regions. In addition, Hallgren and Sourander also reported average iron concentrations for patients between 30 and 100 years of age for the red nucleus (*N* = 44), substantia nigra (*N* = 52), dentate nucleus (*N* = 45), and caudate nucleus (*N* = 58). These average concentrations were also included in our analysis.

Next, we examined the degree to which the age trend in our data set corresponded to that reported by Hallgren and Sourander (1958). These authors observed that the adult age‐related increase in deep gray matter iron was pronounced for the putamen. We derived an estimated iron value in the putamen for each participant, based on their age and the Hallgren and Sourander regression equation, which related adult age to putamen iron.

(1) $$y=14.62\;\left\{1–\exp\;\left(–0.04x\right)\right\}+0.46$$

In Equation (1), *y* is the nonhaemin iron in the putamen (mg/100 g fresh weight), and *x* is the individual age in years. We then correlated the predicted values for our sample, based on the Hallgren and Sourander regression equation for the histologically obtained values, with our susceptibility values derived from QSM. As described in more detail in the Results (Section 3.1 [or 3.2]), the comparison of our susceptibility data to the Hallgren and Sourander histology data demonstrate good validity.

### 2.6 Voxelwise QSM

**FIGURE 1:**
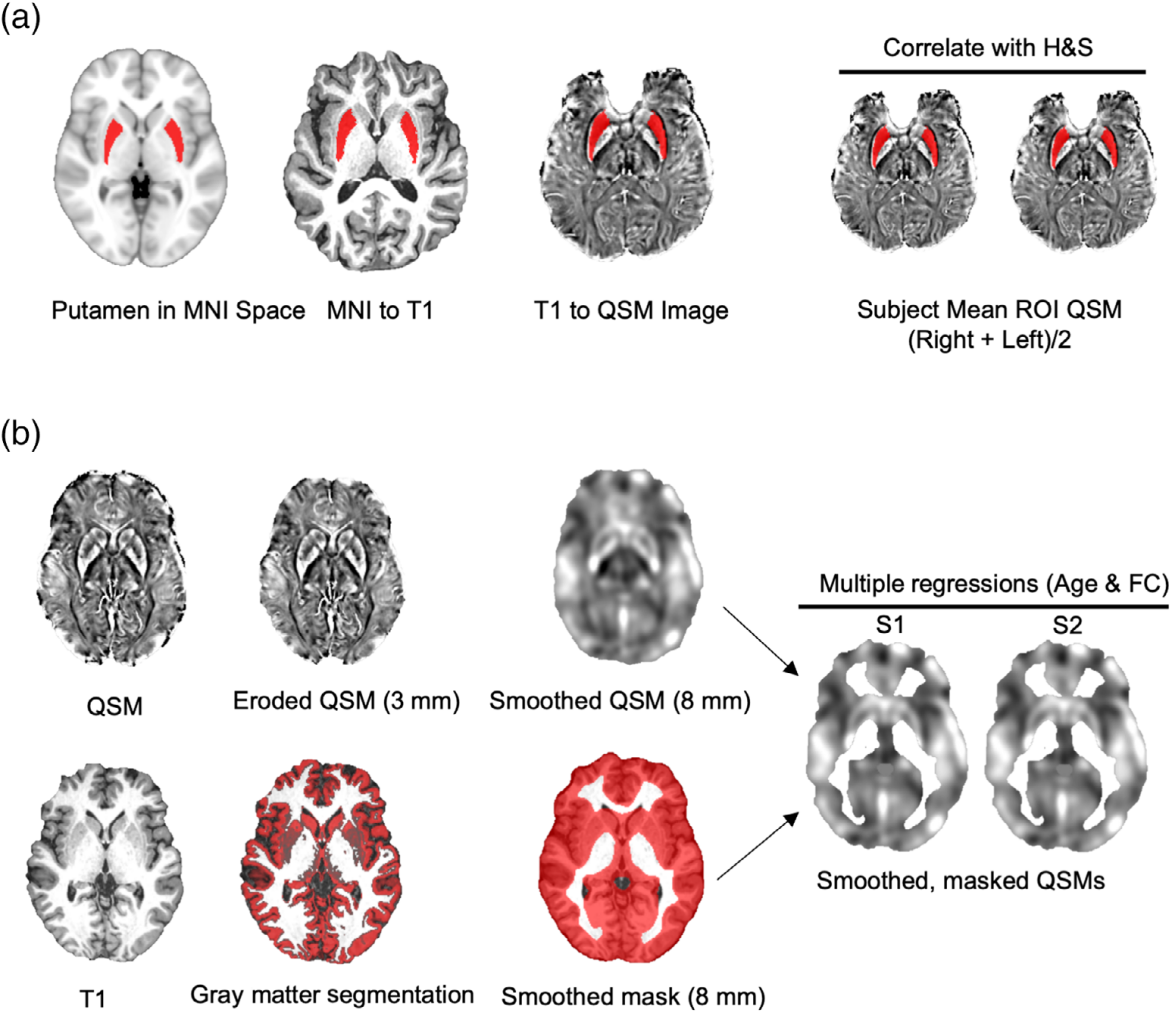
Pipeline for analyses of brain data. (a). Steps for ROI analysis. Representative ROI, T1, and susceptibility images. (b). Steps for voxelwise QSM analyses. Representative QSM, T1, and gray matter segmentation. S1 and S2 represent subjects 1 and 2

We used FAST (FMRIB's Automated Segmentation Tool) to segment the 3D T1‐weighted structural images into gray matter, white matter, and cerebrospinal fluid. Each participant's T1 and reconstructed susceptibility image were coregistered to their average magnitude image. The resulting coregistration file was used to warp the magnitude image to the 3D T1‐weighted structural image using FLIRT in FSL. The 3D T1‐weighted structural image was then registered to the MNI template using FSL FLIRT and warped using FNIRT. The resulting warp transformation parameters were then used to normalize the segmented gray matter images, magnitude images, and reconstructed susceptibility maps for each into MNI space. Visual inspection of the reconstructed susceptibility map revealed oscillatory susceptibility estimation around the edges of the brain. Because this would cause unreliable whole‐brain voxel‐wise analysis, we eroded the binary brain masks (previously generated by the average magnitude images) by 3 mm. We then masked the reconstructed susceptibility images by the binary, eroded brain masks of each participant to ensure reliable analyses. Susceptibility map and gray matter images were smoothed with an 8‐mm full width at half maximum Gaussian kernel. These previously validated parameters were validated in voxelwise structural analyses, including voxelwise QSM (Uchida et al., 2019). The gray matter images were then thresholded to exclude nongray matter voxels with an 80% confidence level, binarized, and then used to mask the susceptibility images. An overlap image was created, representing the number of participants containing gray matter in each voxel. This overlap image was thresholded and used to mask the analytical area such that only voxels that included at least 20% of the participants (*n* = 13) were analyzed. To replicate previous findings indicating increased brain‐iron accumulation in sensory‐motor regions (Acosta‐Cabronero et al., 2016), a multiple regression was performed to identify voxels associated with age. Next, a multiple regression was performed to identify voxels associated with general fluid cognition. The resulting beta images were analyzed using the SPM toolbox. Voxels significant at the uncorrected threshold of *p* <.0001 were cluster corrected at 500 cubic voxels. Voxels that survived family‐wises error rate (FWER) correction at *p* <.05 were also reported (Figure 1).

### 2.7 Mediation and moderation analyses

To determine whether susceptibility mediates the relation between age and general fluid cognition, we performed a mediation analysis with the PROCESS macro (Hayes 2013), implemented in SAS 9.4 (SAS Institute, Inc., Cary, NC). Unlike ROI analyses, voxelwise analyses are not constrained by our definitions of functional or structural areas. To target analyses to those clusters related to fluid cognition and limit the number of multiple comparisons, mediation and moderation analyses were only performed for voxel clusters in which, for all participants combined, susceptibility was correlated negatively with cognition (i.e., higher iron associated with lower cognitive performance). We then calculated, for each participant, the average susceptibility in each cluster. To ensure that collinearity would not negatively impact mediation results, we entered age and susceptibility for the identified clusters into a regression model and calculated the variance inflation factors (VIFs). After removing the two smallest clusters, because they contributed to multicollinearity (VIFs >5), each remaining cluster exhibited VIF <5, confirming that each remaining cluster's susceptibility values represented unique variance.

We then entered the cluster susceptibility values into a mediation model in which age was a predictor (*x*) of fluid cognition (*y*), and each cluster's susceptibility mediated the age and fluid cognition relation. This mediation is a path model in which the relation of age to each of the clusters is a separate *a* path, the relation of each of the clusters to fluid cognition (controlling for age) is a separate *b* path, the relation of age to fluid cognition (the total effect of age) is the *c* path, and the direct effect of age (controlling for all of the cluster mediators) is the *c*′ path. We modeled the mediators as operating in parallel; thus each mediator was covaried for the others. A significant *a × b* path interaction for a mediator variable would imply that the predictor (age) effect on the outcome variable (fluid cognition) is indirect, operating through that variable rather than direct. The *a × b* path interaction effects were tested with bootstrap confidence intervals. To confirm that results were reliable and did not vary due to cluster exclusion for multicollinearity, we also performed separate mediation analyses with each cluster tested as an independent mediator (see Table 4).

Additionally, the moderation effects of age and susceptibility in each cluster were calculated using the PROCESS macro. Finally, for clusters with significant interactions (*p* <.01), we performed a Johnson–Neyman test to identify the particular point, on the age continuum, at which susceptibility predicts fluid cognition (Johnson & Fay, 1950).

## 3 RESULTS

### 3.1 Cognitive assessments

**FIGURE 2:**
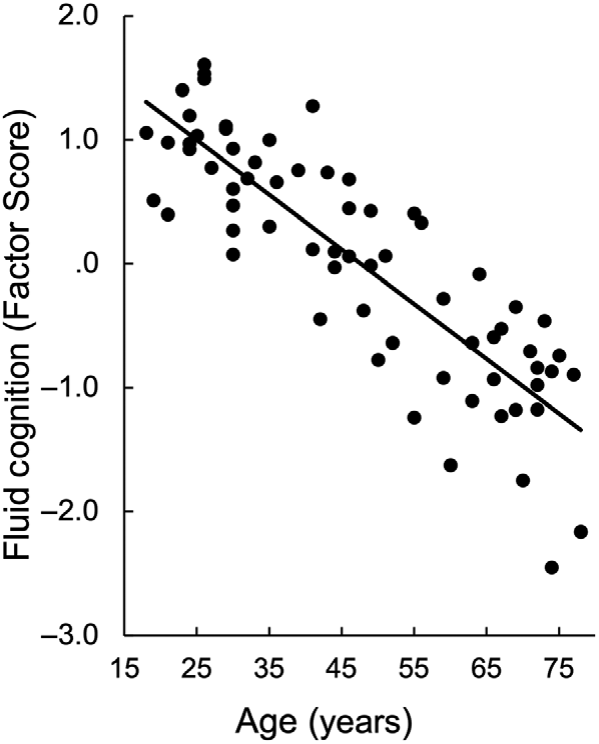
Fluid cognition (*y*‐axis) negatively correlates with age (*x*‐axis), *p* <.0001

Pearson correlations revealed that the three cognitive factor scores were positively correlated with each other (*r* range = .48 to .84, all *p* values <.0001) and negatively correlated with age (*r* range −.79 to −.59, all *p* values <.0001). However, the residual domain scores (i.e., each domain covaried for all tests outside the domain) were not correlated with age (*r* range −.22 to −.03, all *p* values >.05). Thus, we focus on overall fluid cognition factor score that showed a strong negative relation with age, *r*(65) = −.86, *p* <.0001 (Figure 2).

### 3.2 ROI analysis and validation

**FIGURE 3:**
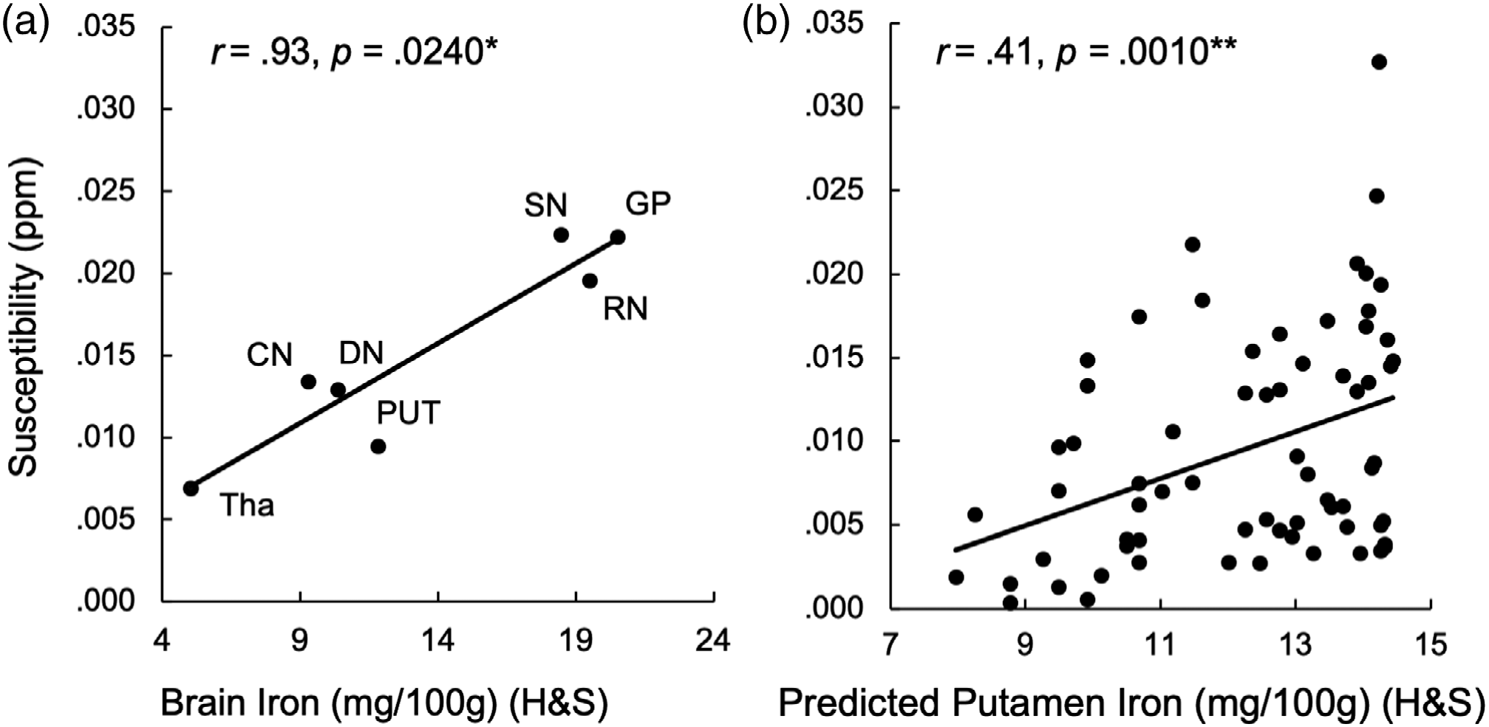
Validation of subcortical susceptibility values with histologically measured brain iron. (a) Average ROI susceptibility (*y*‐axis) significantly correlates with histologically measured brain iron from Hallgren and Sourander (H&S, *x*‐axis). (b) Age‐predicted brain iron (calculated with H&S formula, *x*‐axis) significantly correlates with observed putamen susceptibility (*y*‐axis). ppm indicates parts per million. mg/100 g indicates milligrams of brain iron per 100 g of fresh weight. Level of significance indicated by * (* <.05, ** <.01)

Pearson correlations revealed that average susceptibility in the red nucleus and putamen correlated positively with age after Bonferroni correction for multiple comparisons (*p* <.0056). Age correlations for all other ROIs were not statistically significant (Table 2). For a subset of these ROIs corresponding to those reported by Hallgren and Sourander (1958), a correlation indicated a significant relationship between our susceptibility values and the histologically defined iron (Figure 3a). Finally, our observed putamen susceptibility aligned very closely with the estimated values based on the Hallgren and Sourander age regression equation (Figure 3b).

**TABLE 2:** Susceptibility and age correlation for each of the subcortical brain regions

Region	*r* (age)	*p*	Significance
Substantia nigra	.0437	.7255	ns
Red nucleus	.4840	<.0001	**
Putamen	.3780	.0010	*
Thalamus	.0784	.5283	ns
Globus pallidus	−.3186	.0078	ns
Caudate nucleus	−.0474	.7057	ns
Amygdala	.2538	.0398	ns
Hippocampus	.0300	.8102	ns
Dentate nucleus	.2170	.0778	ns

*Note*: Significance denotes *p* value after Bonferroni correction *p < .05 **p < .01.

### 3.3 Voxelwise QSM

**FIGURE 4:**
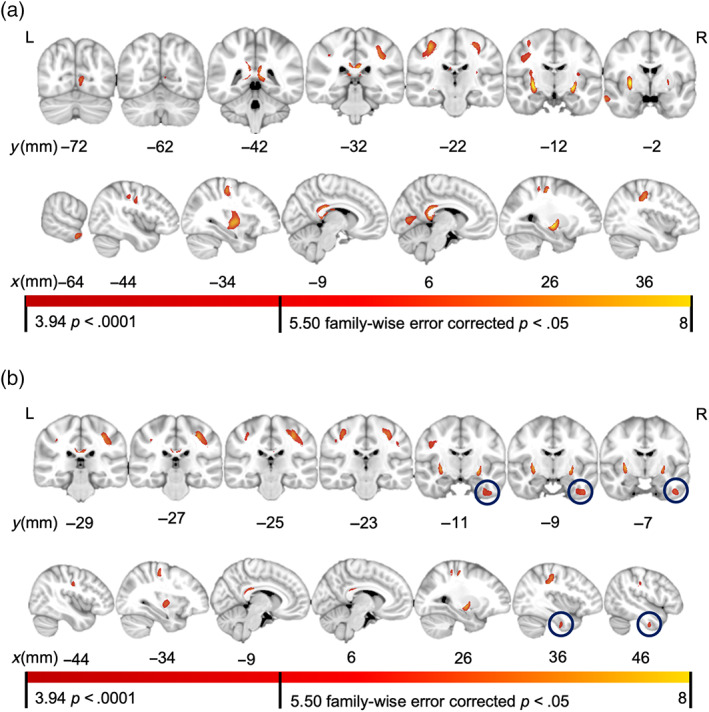
Voxelwise QSM results. (a). Voxels correlated with age. *t*‐values for voxels showing a positive age and susceptibility association. (b). Voxels correlated with fluid cognition. *t*‐values for voxels showing a negative fluid cognition and susceptibility association. All clusters >500 cubic voxels

Voxelwise QSM analyses revealed seven clusters in which susceptibility values were positively associated with age: left putamen, cluster size = 2,965, peak *t*‐value = 8.95; right putamen, cluster size =1,342, peak *t*‐value = 8.03; a cluster spanning the left pre‐ and postcentral gyri, cluster size = 2,863, peak *t*‐value = 6.46; a cluster spanning the right pre‐ and postcentral gyri, cluster size = 2,788, peak *t*‐value = 6.87; bilateral posterior cingulate gyrus, cluster size = 2,863, peak *t*‐value = 8.03; right intra calcarine cortex, cluster size = 941, peak *t*‐value = 5.29; left middle temporal gyrus, cluster size = 541, peak *t*‐value = 5.36 (Figure 4a).

Susceptibility in seven clusters was negatively associated with general fluid cognition: left putamen, cluster size = 1,071, peak *t*‐value = 6.39; right putamen, cluster size =643, peak *t*‐value = 6.17; left precentral gyrus, cluster size = 1,087, peak *t*‐value = 5.67; left postcentral gyrus, cluster size = 587, peak *t*‐value = 4.78; a cluster spanning the right pre‐ and postcentral gyri, cluster size =2,718, peak *t*‐value = 6.19; bilateral posterior cingulate gyrus, cluster size =648, peak *t*‐value = 5.96; right inferior temporal gyrus, cluster size = 747, peak *t*‐value = 4.75 36 (Figure 4b).

### 3.4 Mediation analysis

**FIGURE 5:**
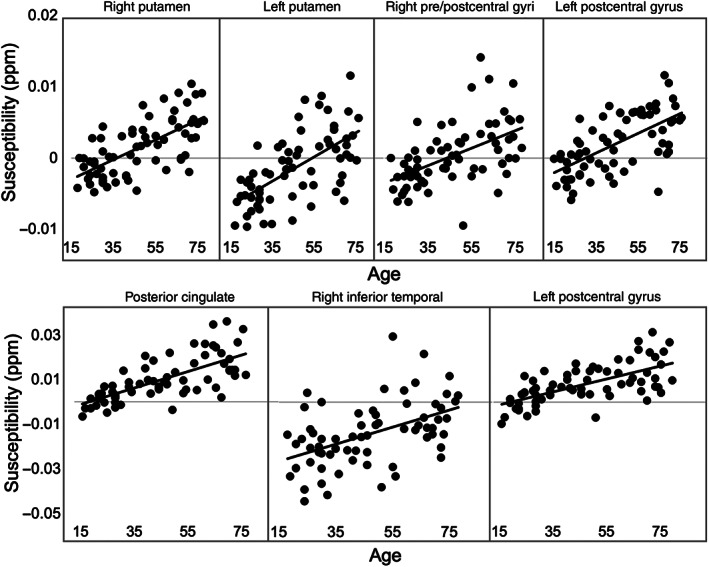
Relation of age (*x*‐axis) to average susceptibility (*y*‐axis) in each identified cluster negatively associated with fluid cognition

The mediation analysis revealed that the effects of age on all of the mediators (*a* paths) were significant (see scatter plots in Figure 5), as well as the total effect of age (*c* path) on overall fluid cognition. Susceptibility in the right inferior temporal gyrus was significantly related to fluid cognition controlling for age (*b* path). Additionally, a significant *a × b* interaction indicated that susceptibility in the right inferior temporal gyrus was a significant mediator of the relation between age and fluid cognition. The mediation effect was significant but partial, in that the direct effect of age remained significant with the mediators taken into account (Tables 3 and 4).

**TABLE 3:** Mediation of age and fluid cognition relation by average cluster susceptibility

Variable 1	Variable 2	Coeff.	*SE*	*t*	*p*	Lower CI	Upper CI
*A path*							
Age	Posterior cingulate	0.0002	<0.0001	6.3771	<.0001	0.0001	0.0002
Age	Right inf. temp.	0.0004	0.0001	4.4181	<.0001	0.0002	0.0006
Age	Left putamen	0.0004	<0.0001	7.9540	<.0001	0.0003	0.0005
Age	Left precentral gyrus	0.0001	<0.0001	7.0122	<.0001	0.0001	0.0002
Age	Right pre/postcentral gyri	0.0001	<0.0001	6.4685	<.0001	0.0001	0.0002
*B path*							
Posterior cingulate	Fluid cognition	12.5779	16.9666	0.7413	.4614	−21.3605	46.5164
Right inf. temp.	Fluid cognition	−10.7759	5.2543	−2.0509	.0447	−21.2861	−0.2657
Left putamen	Fluid cognition	12.3903	8.7736	1.4122	.1631	−5.1597	29.9402
Left precentral gyrus	Fluid cognition	−5.2183	23.4318	0.2227	.8245	−41.6525	52.0892
Right pre/postcentral gyri	Fluid cognition	−32.3488	21.5720	−1.4996	.1390	−75.4995	10.8020

Mediation		Effect	*SE*	*t*	*p*	Lower CI	Upper CI
A × B interaction							
	Posterior cingulate	0.0021	0.0030	—	—	−0.0041	0.0078
	Right inf. temp.	−0.0041	0.0021	—	—	−0.0082	−0.0001
	Left putamen	0.0047	0.0034	—	—	−0.0028	0.0108
	Left precentral gyrus	0.0007	0.0037	—	—	−0.0071	0.0075
	Right pre/postcentral gyri	−0.0046	0.0033	—	—	−0.0111	0.0020
C path	Total effect of age	−0.0441	0.0033	−13.2899	<.0001	−0.0507	−0.0375
C′ path	Direct effect of age	−0.0430	0.0054	−7.9136	<.0001	−0.0538	−0.0321

*Note*: Interaction terms tested using confidence intervals (CIs).

**TABLE 4:** Mediation of age and fluid cognition relation by average cluster susceptibility in individual models

	Variable	Coeff.	*SE*	*t*	*p*	Lower CI	Upper CI
*Left postcentral gyrus*							
A path	Age	0.0001	0	4.8386	<.0001	0.0001	0.0002
B path	Fluid cognition	−23.7464	16.198	−1.466	.1475	−56.1057	8.6129
A × B	Interaction	−0.0029	0.0023	—	—	−0.0079	0.0009
C′ path	Direct effect of age	−0.0412	0.0038	−10.7411	<.0001	−0.0489	−0.0336
*Right putamen*							
A path	Age	0.0003	0	6.8074	<.0001	0.0002	0.0004
B path	Fluid cognition	−2.581	9.1907	−0.2808	.7797	−20.9416	15.7795
A × B	Interaction	−0.0008	0.0024	—	—	−0.0057	0.004
C′ path	Direct effect of age	−0.0433	0.0044	−9.901	<.0001	−0.0521	−0.0346
*Posterior cingulate*							
A path	Age	0.0002	0	6.3771	<.0001	0.0001	0.0002
B path	Fluid cognition	−4.5566	15.722	−0.2898	.7729	−35.965	26.8518
A × B	Interaction	−0.0008	0.0027	—	—	−0.0061	0.0047
C′ path	Direct effect of age	−0.0433	0.0043	−10.1699	<.0001	−0.0519	−0.0348
Right Inf. Temp							
A path	Age	0.0004	0.0001	4.4181	<.0001	0.0002	0.0006
B path	Fluid cognition	−9.8474	4.6406	−2.122	.0377	−19.118	−0.5768
A × B	Interaction	−0.0038	0.0018	—	—	−0.0075	−0.0005
C′ path	Direct effect of age	−0.0404	0.0037	−10.9448	<.0001	−0.0477	−0.033
*Left putamen*							
A path	Age	0.0004	<0.0001	7.954	<.0001	0.0003	0.0005
B path	Fluid cognition	6.3915	8.7166	0.7333	.4661	−11.0221	23.8051
A × B	Interaction	0.0024	0.0032	—	—	−0.0037	0.0089
C′ path	Direct effect of age	−0.0465	0.0047	−9.942	<.0001	−0.0559	−0.0372
*Left precentral gyrus*							
A path	Age	0.0001	<0.0001	7.0122	<.0001	0.0001	0.0002
B path	Fluid cognition	−18.2651	20.7677	−0.8795	.3824	−59.7535	23.2233
A × B	Interaction	−0.0025	0.0029	—	—	−0.0087	0.003
C′ path	Direct effect of age	−0.0416	0.0044	−9.433	<.0001	−0.0504	−0.0328
*Right pre/postcentral gyri*							
A path	Age	0.0001	<0.0001	6.4685	<.0001	0.0001	0.0002
B path	Fluid cognition	−32.3553	18.6348	−1.7363	.0873	−69.5829	4.8722
A × B	Interaction	−0.0046	0.0027	—	—	−0.0100	0.0006
C′ path	Direct effect of age	−0.0396	0.0042	−9.4388	<.0001	−0.0479	−0.0312

*Note*: Interaction terms tested using confidence intervals (CIs).

**FIGURE 6:**
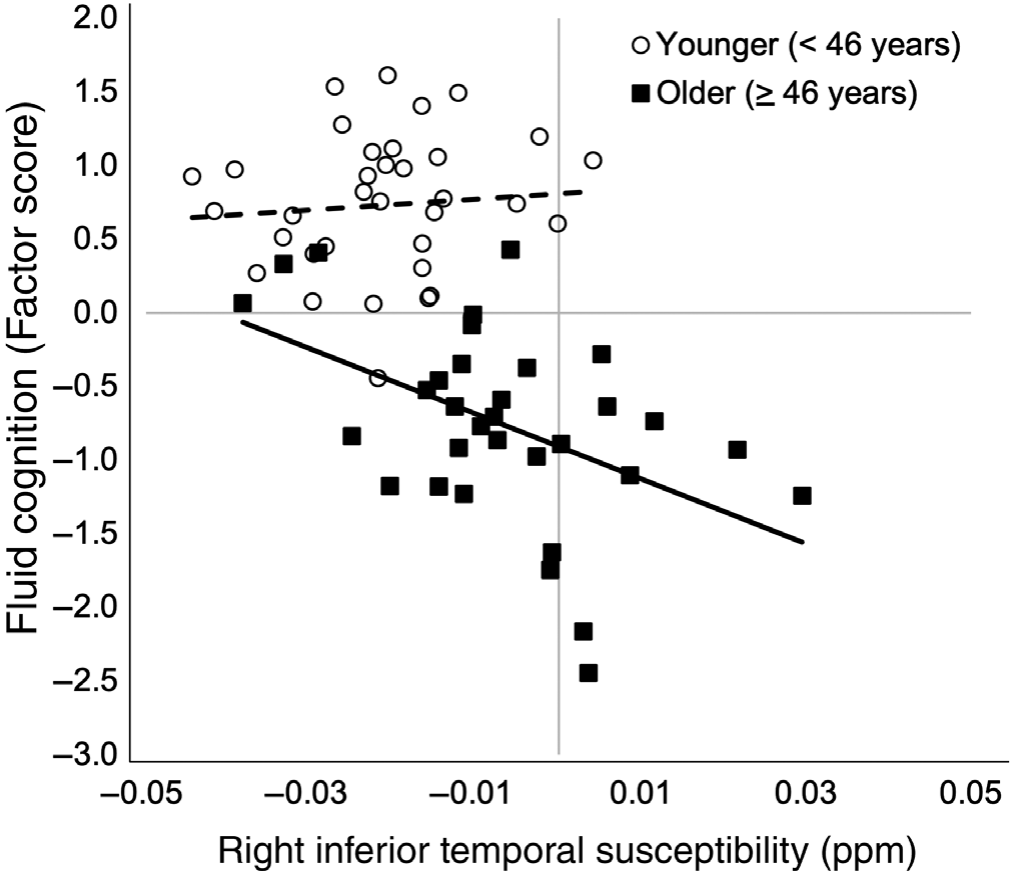
Visualization of age X right inferior temporal susceptibility (in parts per million). Two age groups plotted as defined by the Johnson–Neyman test (older adult aged 46 years and greater). Solid black boxes and trend line indicate older adult right inferior temporal susceptibility (*x*‐axis) plotted against older adult fluid cognition (*y*‐axis), transparent circles, and dashed line indicate younger adult susceptibility and fluid cognition

Moderation analyses reveal that inferior temporal gyrus susceptibility interacted with age, *F*(1, 63) = 7.27, *p* = .009, *R* ^2^_change_ = .026. To better understand the nature of the interaction, we performed a Johnson–Neyman test to identify the age at which the inferior temporal gyrus susceptibility negatively predicted fluid cognition. The results revealed that inferior temporal gyrus susceptibility negatively predicted fluid cognition at 46 years of age and above (*p* <.05) (Figure 6). There were no significant moderation effects for any other cluster (all *p*‐values >.05). Although global gray matter volumes did not significantly correlate with global QSM values (*r* = −.173, *p* = .162), we performed post hoc analyses to determine if our effects were attributable to regional gray matter volume. We added gray matter volumes of the significant right inferior temporal cluster were added to the mediation and moderation models as covariates. In these models the effects for both mediation (see Table S1) and moderation, *F*(1, 62) = 7.40, *p* = .009, *R* ^2^_change_ = .027, remained significant.

## 4 DISCUSSION

Utilizing both ROI and voxelwise approaches, this study replicates previous findings that iron in both deep gray matter and frontal motor regions accumulates with age (Acosta‐Cabronero et al., 2016; Zachariou et al., 2020). This study also presents the novel finding that iron in several other cortical regions, including the postcentral gyri, posterior cingulate, and middle and inferior temporal gyri, are associated with age and fluid cognition. In particular, iron within the right inferior temporal cortex was significant mediator of age‐related decline in fluid cognition, such that increasing iron in this region was associated with decreasing cognition, particularly for individuals beyond 45 years of age (Figure 6).

Results from our ROI analysis show that QSM, a relatively recent MRI technique, is a valid measure for estimating brain iron. We were able to validate the striatal distribution across regions (Figure 3a) and the histology‐predicted degree of age‐related increase of iron in the putamen (Figure 3b). While in‐vivo estimates of brain iron using the ROI approach have elucidated its relation to cognition (Daugherty et al., 2015; Penke et al., 2012; Sullivan et al., 2009; Van Bergen et al., 2018; Zachariou et al., 2020), our results suggest that the voxelwise approach, which is not constrained to predefined areas or averaging over large cortical structures, reveals novel relations.

### 4.1 Age‐related brain iron accumulation

Age‐related iron accumulation in the putamen and sensorimotor regions is well established in previous findings that utilize various iron‐measurement techniques, including histology (Hallgren & Sourander, 1958; Ramos et al., 2014), in vivo ROI (Daugherty et al., 2015), and whole‐brain analyses (Acosta‐Cabronero et al., 2016). In line with these findings, our age‐related, voxelwise QSM analysis found that iron was positively associated with age in several sensorimotor regions (putamen, sensorimotor cortices, and the cingulate gyrus). We also found age‐related iron deposition in nonmotor areas (visual cortex and middle temporal gyrus) (Figure 4a). The detection of cortical regions outside sensory areas suggests an advantage of the current whole‐brain approach, revealing novel relations beyond predefined ROIs. However, it should be noted that the current approach has some limitations. In particular, the variability of QSM susceptibility in some structures, such as the hippocampus (Daugherty et al., 2015; Rodrigue, Daugherty, Haacke, & Raz, 2013), may mask age‐related effects, especially for whole‐brain, non‐ROI based approaches (Acosta‐Cabronero et al., 2016).

### 4.2 Brain iron accumulation relates to fluid cognition

The fluid cognition‐related voxelwise QSM analysis found that iron was negatively associated with fluid cognition in many regions related to age (putamen, sensorimotor cortices, and cingulate gyrus) and one cluster that we did not detect in the age‐related results (right inferior temporal gyrus). Since fluid cognition highly negatively correlates with age (Figure 4b), the overlapping results are not surprising. The mediation analysis revealed that brain iron in the right inferior temporal gyrus cluster was associated positively with age *p* = .0001 (Table 3). Therefore, we suspect that we did not find this result in the age‐related voxelwise analysis due to cluster thresholding. Indeed, relaxing the cluster‐size threshold from 500 mm^3^ to 450 mm^3^ reveals an age‐related cluster in the right inferior temporal gyrus (cluster size = 476, peak *t* = 4.69). Further relaxing cluster thresholds also yielded significant clusters in the superior temporal lobe, parahippocampus, cerebellum, and left inferior temporal gyrus. We recognize that voxelwise approaches to structural data have the risk of false negatives (Whitwell, 2009), and that cluster thresholding, while necessary to avoid spurious results, can be arbitrary and mask effects in small clusters.

### 4.3 Significance of the inferior temporal gyri

While we expected a negative relation between fluid cognition and brain iron, we did not predict that fluid cognition would specifically relate to brain iron in the inferior temporal gyrus. Previous ROI studies have indicated iron in striatal regions, including the putamen, is predictive of dementia ratings (Sullivan et al., 2009), verbal working memory (Daugherty et al., 2015), and motor function (Daugherty et al., 2015). Our fluid cognition measure indexes speed‐dependent cognitive functions within three domains: executive function, processing speed, and memory. Researchers have noted that age‐related changes in sensory degradation and processing speed slowing explain much of the shared age‐related variance between cognitive domains and may cause age‐related cognitive declines (Baltes & Lindenberger, 1997; Monge & Madden, 2016; Schneider & Pichora‐Fuller, 2000). Thus, we expected that brain iron in primary visual and sensorimotor regions would mediate the age‐cognition relation. While we did observe that visual and sensorimotor areas accumulate iron with age, iron in these regions does not mediate the relationship between age and fluid cognition. One possibility for this discrepancy is that brain iron mediation effects in these regions may be more subtle or only present for older adults. Future studies with a larger sample of the older adult population should attempt to replicate these findings.

Previous studies have reported increased inferior temporal iron in patients with Alzheimer's disease (Tao et al., 2014). The increase of iron in Alzheimer's disease patients has been found to interact with increased neuropathology (e.g., neurofibrillary tangles) and predict cognitive decline rate and magnitude (Ayton et al., 2019). Since the current study did not measure these neuropathologies, we cannot rule out that they could mediate our findings. However, all the present participants exhibited MMSE scores of 27 and above (Table 1), so the presence of undetected Alzheimer's disease is unlikely.

The mechanism specific to the inferior temporal cortex that causes brain iron to impact cognition in aging or neuropathology remains unknown. As discussed previously, iron accumulation may contribute to cell death (Block, Zecca, & Hong, 2007) or disrupt neurotransmission (Becerril‐Ortega et al., 2014; Zecca et al., 2004). The exact mechanism responsible for the decline in fluid cognition may be related to the specific cognitive domains and functions in this cortical region. For example, prevalent cell death may lead to degraded object representation, and disrupted neurotransmission may have broader implications for processes involved in memory storage and attentional control. Iron accumulation has been linked to decreased gray matter volumes (Penke et al., 2012; Sullivan et al., 2009) and functional connectivity (Salami, Avelar‐Pereira, Garzón, Sitnikov, & Kalpouzos, 2018; Zachariou et al., 2020).

Importantly, it is unlikely that true effects are lateralized, and that the right inferior temporal cortex cluster is the only region in which brain iron acts as a mediator. As previously noted, voxelwise approaches to structural data risk false negatives, and cluster thresholding can be arbitrary and mask effects in small clusters. In fact, a sub‐threshold cluster (200 mm^3^) in the left inferior temporal cortex also exhibited a negative relation to fluid cognition (see Figure S1), and with confidence intervals relaxed to 92.5, mediation of the age‐fluid cognition relation was also significant for this left inferior temporal region (Table 5). Future studies should also probe how brain iron in the inferior temporal cortex and other regions interacts with other determinants of age‐related declines in fluid cognition, such as white matter integrity and functional connectivity.

**TABLE 5:** Mediation of age and fluid cognition relation by average susceptibility in the left inferior temporal cluster

	Variable	Coeff.	*SE*	*t*	*p*	Lower CI	Upper CI
*Left inferior temporal cortex*							
A path	Age	0.0004	0.0001	4.0379	<.0001	0.0002	0.0005
B path	Fluid cognition	−8.7759	4.5479	−1.9297	.0581	−17.0074	−0.5445
A × B	Interaction	−0.0031	0.0019	—	—	−0.0070	−0.0002
C′ path	Direct effect of age	−0.0410	0.0036	−11.2651	<.0001	−0.0476	−0.0344

*Note*: Interaction terms tested using confidence intervals (CIs) of 0.925.

### 4.4 Brain iron as a biomarker

Previous studies have suggested that brain iron accumulation may be a biomarker of mild cognitive impairment (Sun et al., 2017). Although previous work targeted brain iron in subcortical regions, the present findings demonstrated that iron in supratentorial cortical regions, in this case inferior temporal cortex, may have a causal role in cognitive decline in the context of healthy aging. Additionally, we found that increasing iron in the right inferior temporal cortex predicts decreasing fluid cognition beyond 45 years of age. Data from fMRI also suggest that middle adulthood is a period during which a decline in functional connectivity in visual sensory regions contributes to age‐related differences in task‐related brain activation (Madden et al., 2017b; Monge et al., 2017). Thus, like declines in functional connectivity, brain iron accumulation is detectable relatively early in healthy aging and neurodegenerative disease and has predictive potential. However, the cross‐sectional nature of the current study limits our conclusions regarding age‐specificity. Longitudinal studies tracking brain iron in conjunction with age‐related cognitive decline and MCI onset should lend further insights.

## 5 CONCLUSIONS

This study demonstrates that QSM is a valid measure of brain iron that is useful for understanding the interaction of adult age, iron, and cognition. Here, we found that increasing brain iron in the right inferior temporal cortex contributed to increased decline in fluid cognition, for individuals beyond 45 years of age. Iron within the left inferior temporal cortex exhibited a similar pattern, though at a lower statistical threshold. These novel findings suggest that iron may have a mediating role in cognitive decline, is a critical source of variance in cognitive aging studies, and may be a biomarker of neurodegenerative disease.

## CONFLICT OF INTEREST

The authors declare no potential conflict of interest.

## Supporting information


**Figure S1** Voxels correlated with fluid cognition including the left inferior temporal cortex. *t*‐values for voxels showing a negative fluid cognition and susceptibility association. Blue circles highlight the left inferior temporal cortex

**Table S1** Mediation of age and fluid cognition relation by average cluster susceptibility in the right inferior temporal cortex (RITC) controlling for gray matter volume

## Data Availability

All behavioral and brain data that support the findings of this study are available upon reasonable request to the corresponding author.

## References

[hbm25706-bib-0001] Acosta‐Cabronero, J. , Betts, M. J. , Cardenas‐Blanco, A. , Yang, S. , & Nestor, P. J. (2016). In vivo MRI mapping of brain iron deposition across the adult lifespan. Journal of Neuroscience, 36, 364–374. 10.1523/JNEUROSCI.1907-15.2016 26758829 PMC4710766

[hbm25706-bib-0002] Acosta‐Cabronero, J. , Williams, G. B. , Cardenas‐Blanco, A. , Arnold, R. J. , Lupson, V. , & Nestor, P. J. (2013). In vivo quantitative susceptibility mapping (QSM) in Alzheimer's disease. PLoS One, 8, e81093. 10.1371/journal.pone.0081093 24278382 PMC3836742

[hbm25706-bib-0003] Ayton, S. , Fazlollahi, A. , Bourgeat, P. , Raniga, P. , Ng, A. , Lim, Y. Y. , … Bush, A. I. (2017). Cerebral quantitative susceptibility mapping predicts amyloid‐beta‐related cognitive decline. Brain, 140, 2112–2119. 10.1093/brain/awx137 28899019

[hbm25706-bib-0004] Ayton, S. , Lei, P. , & Bush, A. I. (2013). Metallostasis in Alzheimer's disease. Free Radical Biology and Medicine, 62, 76–89. 10.1016/j.freeradbiomed.2012.10.558 23142767

[hbm25706-bib-0005] Ayton, S. , Wang, Y. , Diouf, I. , Schneider, J. A. , Brockman, J. , Morris, M. C. , & Bush, A. I. (2019). Brain iron is associated with accelerated cognitive decline in people with Alzheimer pathology. Molecular Psychiatry, 1‐10, 456–460. 10.1136/jnnp-2017-316551 PMC6698435 30778133

[hbm25706-bib-0006] Baltes, P. B. , & Lindenberger, U. (1997). Emergence of a powerful connection between sensory and cognitive functions across the adult life span: A new window to the study of cognitive aging? Psychology and Aging, 12, 12–21. 10.1037/0882-7974.12.1.12 9100264

[hbm25706-bib-0007] Bartzokis, G. , Beckson, M. , Hance, D. B. , Marx, P. , Foster, J. A. , & Marder, S. R. (1997). MR evaluation of age‐related increase of brain iron in young adult and older normal males. Magnetic Resonance Imaging, 15, 29–35. 10.1016/S0730-725X(96)00234-2 9084022

[hbm25706-bib-0008] Bartzokis, G. , Tishler, T. A. , Shin, I. S. , Lu, P. H. , & Cummings, J. L. (2004). Brain ferritin iron as a risk factor for age at onset in neurodegenerative diseases. Annals of the New York Academy of Sciences, 1012, 224–236. 10.1196/annals.1306.019 15105269

[hbm25706-bib-0009] Becerril‐Ortega, J. , Bordji, K. , Fréret, T. , Rush, T. , & Buisson, A. (2014). Iron overload accelerates neuronal amyloid‐β production and cognitive impairment in transgenic mice model of Alzheimer's disease. Neurobiology of Aging, 35, 2288–2301. 10.1016/j.neurobiolaging.2014.04.019 24863668

[hbm25706-bib-0010] Beck, A. (1978). The Beck depression inventory. San Antonio, TX: Psychological Corporation.

[hbm25706-bib-0011] Block, M. L. , Zecca, L. , & Hong, J.‐S. (2007). Microglia‐mediated neurotoxicity: Uncovering the molecular mechanisms. Nature Reviews Neuroscience, 8, 57–69. 10.1038/nrn2038 17180163

[hbm25706-bib-0012] Bush, A. I. (2013). The metal theory of Alzheimer's disease. Journal of Alzheimer's Disease, 33(Suppl 1), S277–S281. 10.3233/JAD-2012-129011 22635102

[hbm25706-bib-0013] Cherny, R. A. , Atwood, C. S. , Xilinas, M. E. , Gray, D. N. , Jones, W. D. , McLean, C. A. , … Kim, Y.‐S. (2001). Treatment with a copper‐zinc chelator markedly and rapidly inhibits β‐amyloid accumulation in Alzheimer's disease transgenic mice. Neuron, 30, 665–676. 10.1016/S0896-6273(01)00317-8 11430801

[hbm25706-bib-0014] Cornett, C. , Markesbery, W. , & Ehmann, W. (1998). Imbalances of trace elements related to oxidative damage in Alzheimer's disease. Neurotoxicology, 19(3), 339–345. 9621340

[hbm25706-bib-0015] Craik, F. I. M. , & Salthouse, T. A. (2000). The handbook of aging and cognition (2nd ed.). Mahwah, NJ: Erlbaum.

[hbm25706-bib-0016] Dagley, A. , LaPoint, M. , Huijbers, W. , Hedden, T. , McLaren, D. G. , Chatwal, J. P. , … Rentz, D. M. (2017). Harvard aging brain study: Dataset and accessibility. NeuroImage, 144, 255–258. 10.1016/j.neuroimage.2015.03.069 25843019 PMC4592689

[hbm25706-bib-0017] Daugherty, A. M. , Haacke, E. M. , & Raz, N. (2015). Striatal iron content predicts its shrinkage and changes in verbal working memory after two years in healthy adults. Journal of Neuroscience, 35, 6731–6743. 10.1523/JNEUROSCI.4717-14.2015 25926451 PMC4412893

[hbm25706-bib-0018] Folstein, M. F. , Folstein, S. E. , & Mchugh, P. R. (1975). Mini‐mental state—practical method for grading cognitive state of patients for clinician. Journal of Psychiatric Research, 12, 189–198. 10.1016/0022-3956(75)90026-6 1202204

[hbm25706-bib-0019] Gershon, R. C. , Wagster, M. V. , Hendrie, H. C. , Fox, N. A. , Cook, K. F. , & Nowinski, C. J. (2013). NIH toolbox for assessment of neurological and behavioral function. Neurology, 80, S2–S6. 10.1212/WNL.0b013e3182872e5f 23479538 PMC3662335

[hbm25706-bib-0020] Ghassaban, K. , Liu, S. , Jiang, C. , & Haacke, E. M. (2019). Quantifying iron content in magnetic resonance imaging. NeuroImage, 187, 77–92. 10.1016/j.neuroimage.2018.04.047 29702183

[hbm25706-bib-0021] Gotz, M. E. , Double, K. , Gerlach, M. , Youdim, M. B. , & Riederer, P. (2004). The relevance of iron in the pathogenesis of Parkinson's disease. Annals of the New York Academy of Sciences, 1012, 193–208. 10.1196/annals.1306.017 15105267

[hbm25706-bib-0022] Grossi, C. , Francese, S. , Casini, A. , Rosi, M. C. , Luccarini, I. , Fiorentini, A. , … Casamenti, F. (2009). Clioquinol decreases amyloid‐β burden and reduces working memory impairment in a transgenic mouse model of Alzheimer's disease. Journal of Alzheimer's Disease, 17, 423–440. 10.3233/JAD-2009-1063 19363260

[hbm25706-bib-0023] Gutteridge, J. M. C. (1992). Iron and oxygen radicals in brain. Annals of Neurology, 32, S16–S21. 10.1002/ana.410320705 1510375

[hbm25706-bib-0024] Haacke, E. M. , Cheng, N. Y. , House, M. J. , Liu, Q. , Neelavalli, J. , Ogg, R. J. , … Obenaus, A. (2005). Imaging iron stores in the brain using magnetic resonance imaging. Magnetic Resonance Imaging, 23, 1–25. 10.1016/j.mri.2004.10.001 15733784

[hbm25706-bib-0025] Hallgren, B. , & Sourander, P. (1958). The effect of age on the non‐haemin iron in the human brain. Journal of Neurochemistry, 3, 41–51. 10.1111/j.1471-4159.1958.tb12607.x 13611557

[hbm25706-bib-0026] Hare, D. , Ayton, S. , Bush, A. , & Lei, P. (2013). A delicate balance: Iron metabolism and diseases of the brain. Frontiers in Aging Neuroscience, 5(34), 1–19. 10.3389/fnagi.2013.00034 23874300 PMC3715022

[hbm25706-bib-0027] He, N. , Ling, H. , Ding, B. , Huang, J. , Zhang, Y. , Zhang, Z. , … Yan, F. (2015). Region‐specific disturbed iron distribution in early idiopathic Parkinson's disease measured by quantitative susceptibility mapping. Human Brain Mapping, 36, 4407–4420. 10.1002/hbm.22928 26249218 PMC6869507

[hbm25706-bib-0028] Hedden, T. , Mormino, E. C. , Amariglio, R. E. , Younger, A. P. , Schultz, A. P. , Becker, J. A. , … Rentz, D. M. (2012). Cognitive profile of amyloid burden and white matter hyperintensities in cognitively normal older adults. Journal of Neuroscience, 32, 16233–16242. 10.1523/Jneurosci.2462-12.2012 23152607 PMC3523110

[hbm25706-bib-0029] Hedden, T. , Schultz, A. P. , Rieckmann, A. , Mormino, E. C. , Johnson, K. A. , Sperling, R. A. , & Buckner, R. L. (2014). Multiple brain markers are linked to age‐related variation in cognition. Cerebral Cortex, 26, 1388–1400. 10.1093/cercor/bhu238 25316342 PMC4785941

[hbm25706-bib-0030] Ibach, B. , Haen, E. , Marienhagen, J. , & Hajak, G. (2005). Clioquinol treatment in familiar early onset of Alzheimer's disease. Pharmacopsychiatry, 38, 178–179. 10.1055/s-2005-871241 16025421

[hbm25706-bib-0031] Jenkinson, M. , Beckmann, C. F. , Behrens, T. E. , Woolrich, M. W. , & Smith, S. M. (2012). FSL. NeuroImage, 62, 782–790. 10.1016/j.neuroimage.2011.09.015 21979382

[hbm25706-bib-0032] Johnson, P. O. , & Fay, L. C. (1950). The Johnson‐Neyman technique, its theory and application. Psychometrika, 15, 349–367. 10.1007/BF02288864 14797902

[hbm25706-bib-0033] Khattar, N. , Triebswetter, C. , Kiely, M. , Ferrucci, L. , Resnick, S. M. , Spencer, R. G. , & Bouhrara, M. (2021). Investigation of the association between cerebral iron content and myelin content in normative aging using quantitative magnetic resonance neuroimaging. NeuroImage, 239, 118267. 10.1016/j.neuroimage.2021.118267 34139358 PMC8370037

[hbm25706-bib-0034] Koeppen, A. H. (1995). The history of iron in the brain. Journal of the Neurological Sciences, 134(Suppl), 1–9. 10.1016/0022-510X(95)00202-D 8847538

[hbm25706-bib-0035] Langkammer, C. , Schweser, F. , Krebs, N. , Deistung, A. , Goessler, W. , Scheurer, E. , … Fazekas, F. (2012). Quantitative susceptibility mapping (QSM) as a means to measure brain iron? A post mortem validation study. NeuroImage, 62, 1593–1599. 10.1016/j.neuroimage.2012.05.049 22634862 PMC3413885

[hbm25706-bib-0036] Li, K. , & Reichmann, H. (2016). Role of iron in neurodegenerative diseases. Journal of Neural Transmission, 123, 389–399. 10.1007/s00702-016-1508-7 26794939

[hbm25706-bib-0037] Li, W. , Langkammer, C. , Chou, Y. H. , Petrovic, K. , Schmidt, R. , Song, A. W. , … Liu, C. (2015). Association between increased magnetic susceptibility of deep gray matter nuclei and decreased motor function in healthy adults. NeuroImage, 105, 45–52. 10.1016/j.neuroimage.2014.10.009 25315786 PMC4339282

[hbm25706-bib-0038] Li, W. , Wang, N. , Yu, F. , Han, H. , Cao, W. , Romero, R. , … Liu, C. (2015). A method for estimating and removing streaking artifacts in quantitative susceptibility mapping. NeuroImage, 108, 111–122. 10.1016/j.neuroimage.2014.12.043 25536496 PMC4406048

[hbm25706-bib-0039] Li, W. , Wu, B. , Batrachenko, A. , Bancroft‐Wu, V. , Morey, R. A. , Shashi, V. , … Liu, C. (2014). Differential developmental trajectories of magnetic susceptibility in human brain gray and white matter over the lifespan. Human Brain Mapping, 35, 2698–2713. 10.1002/hbm.22360 24038837 PMC3954958

[hbm25706-bib-0040] Li, W. , Wu, B. , & Liu, C. (2011). Quantitative susceptibility mapping of human brain reflects spatial variation in tissue composition. NeuroImage, 55, 1645–1656. 10.1016/j.neuroimage.2010.11.088 21224002 PMC3062654

[hbm25706-bib-0041] Liu, C. , Li, W. , Tong, K. A. , Yeom, K. W. , & Kuzminski, S. (2015). Susceptibility‐weighted imaging and quantitative susceptibility mapping in the brain. Journal of Magnetic Resonance Imaging, 42, 23–41. 10.1002/jmri.24768 25270052 PMC4406874

[hbm25706-bib-0042] Madden, D. J. , Parks, E. L. , Tallman, C. W. , Boylan, M. A. , Hoagey, D. A. , Cocjin, S. B. , … Chen, N. K. (2017a). Frontoparietal activation during visual conjunction search: Effects of bottom‐up guidance and adult age. Human Brain Mapping, 38, 2128–2149. 10.1002/hbm.23509 28052456 PMC5342935

[hbm25706-bib-0043] Madden, D. J. , Parks, E. L. , Tallman, C. W. , Boylan, M. A. , Hoagey, D. A. , Cocjin, S. B. , … Diaz, M. T. (2017b). Sources of disconnection in neurocognitive aging: Cerebral white‐matter integrity, resting‐state functional connectivity, and white‐matter hyperintensity volume. Neurobiology of Aging, 54, 199–213. 10.1016/j.neurobiolaging.2017.01.027 28389085 PMC5401777

[hbm25706-bib-0044] Monge, Z. A. , Geib, B. R. , Siciliano, R. E. , Packard, L. E. , Tallman, C. W. , & Madden, D. J. (2017). Functional modular architecture underlying attentional control in aging. NeuroImage, 155, 257–270. 10.1016/j.neuroimage.2017.05.002 28476664 PMC5512538

[hbm25706-bib-0045] Monge, Z. A. , & Madden, D. J. (2016). Linking cognitive and visual perceptual decline in healthy aging: The information degradation hypothesis. Neuroscience & Biobehavioral Reviews, 69, 166–173. 10.1016/j.neubiorev.2016.07.031 27484869 PMC5030166

[hbm25706-bib-0046] Morris, C. , Candy, J. , Oakley, A. , Bloxham, C. , & Edwardson, J. (1992). Histochemical distribution of non‐haem iron in the human brain. Cells, Tissues, Organs, 144, 235–257. 10.1159/000147312 1529678

[hbm25706-bib-0047] Penke, L. , Hernandéz, M. C. V. , Maniega, S. M. , Gow, A. J. , Murray, C. , Starr, J. M. , … Wardlaw, J. M. (2012). Brain iron deposits are associated with general cognitive ability and cognitive aging. Neurobiology of Aging, 33, 510–517. 10.1016/j.neurobiolaging.2010.04.032 20542597

[hbm25706-bib-0048] Ramos, P. , Santos, A. , Pinto, N. R. , Mendes, R. , Magalhães, T. , & Almeida, A. (2014). Iron levels in the human brain: A post‐mortem study of anatomical region differences and age‐related changes. Journal of Trace Elements in Medicine and Biology, 28, 13–17. 10.1016/j.jtemb.2013.08.001 24075790

[hbm25706-bib-0049] Raz, N. , & Daugherty, A. M. (2018). Pathways to brain aging and their modifiers: Free‐radical‐induced energetic and neural decline in senescence (FRIENDS) model‐A mini‐review. Gerontology, 64, 49–57. 10.1159/000479508 28858861 PMC5828941

[hbm25706-bib-0050] Reitan, R. M. (1986). Trail making test: Manual for administration and scoring. Mesa, AZ: Reitan Neuropsychology Laboratory.

[hbm25706-bib-0051] Ritchie, C. W. , Bush, A. I. , Mackinnon, A. , Macfarlane, S. , Mastwyk, M. , MacGregor, L. , … Tammer, A. (2003). Metal‐protein attenuation with iodochlorhydroxyquin (clioquinol) targeting Aβ amyloid deposition and toxicity in Alzheimer disease: A pilot phase 2 clinical trial. Archives of Neurology, 60, 1685–1691. 10.1001/archneur.60.12.1685 14676042

[hbm25706-bib-0052] Rodrigue, K. M. , Daugherty, A. M. , Haacke, E. M. , & Raz, N. (2013). The role of hippocampal iron concentration and hippocampal volume in age‐related differences in memory. Cerebral Cortex, 23, 1533–1541. 10.1093/cercor/bhs139 22645251 PMC3673172

[hbm25706-bib-0053] Rouault, T. A. , & Cooperman, S. (2006). Brain iron metabolism. Seminars in Pediatric Neurology, 13, 142–148. 10.1016/j.spen.2006.08.002 17101452

[hbm25706-bib-0054] Salami, A. , Avelar‐Pereira, B. , Garzón, B. , Sitnikov, R. , & Kalpouzos, G. (2018). Functional coherence of striatal resting‐state networks is modulated by striatal iron content. NeuroImage, 183, 495–503. 10.1016/j.neuroimage.2018.08.036 30125714

[hbm25706-bib-0055] Salthouse, T. A. (1992). Mechanisms of age‐cognition relations in adulthood. Mahwah, NJ: Erlbaum.

[hbm25706-bib-0056] Salthouse, T. A. (1996). The processing‐speed theory of adult age differences in cognition. Psychological Review, 103, 403–428. 10.1037/0033-295x.103.3.403 8759042

[hbm25706-bib-0057] Salthouse, T. A. , Habeck, C. , Razlighi, Q. , Barulli, D. , Gazes, Y. , & Stern, Y. (2015). Breadth and age‐dependency of relations between cortical thickness and cognition. Neurobiology of Aging, 36, 3020–3028. 10.1016/j.neurobiolaging.2015.08.011 26356042 PMC4609615

[hbm25706-bib-0058] Salthouse, T. A. , & Madden, D. J. (2007). Information processing speed and aging. In J. Deluca & J. Kalmar (Eds.), Information processing speed in clinical populations (pp. 221–241). East Sussex, England: Psychology Press.

[hbm25706-bib-0059] Saults, J. S. , & Cowan, N. (2007). A central capacity limit to the simultaneous storage of visual and auditory arrays in working memory. Journal of Experimental Psychology: General, 136, 663–684. 10.1037/0096-3445.136.4.663 17999578 PMC2621445

[hbm25706-bib-0060] Schneider, B. A. , & Pichora‐Fuller, M. K. (2000). Implications of perceptual deterioration for cognitive aging research. In F. I. M. Craik & T. A. Salthouse (Eds.), The handbook of aging and cognition (2nd ed., pp. 155–219). Mahwah, NJ: Erlbaum.

[hbm25706-bib-0061] Schubert, D. , & Chevion, M. (1995). The role of iron in beta amyloid toxicity. Biochemical and Biophysical Research Communications, 216, 702–707. 10.1006/bbrc.1995.2678 7488167

[hbm25706-bib-0062] Sofic, E. , Paulus, W. , Jellinger, K. , Riederer, P. , & Youdim, M. (1991). Selective increase of iron in substantia nigra zona compacta of parkinsonian brains. Journal of Neurochemistry, 56, 978–982. 10.1111/j.1471-4159.1991.tb02017.x 1704426

[hbm25706-bib-0063] Sullivan, E. V. , Adalsteinsson, E. , Rohlfing, T. , & Pfefferbaum, A. (2009). Relevance of iron deposition in deep gray matter brain structures to cognitive and motor performance in healthy elderly men and women: Exploratory findings. Brain Imaging and Behavior, 3, 167–175. 10.1007/s11682-008-9059-7 20161183 PMC2727611

[hbm25706-bib-0064] Sun, H. , Walsh, A. J. , Lebel, R. M. , Blevins, G. , Catz, I. , Lu, J.‐Q. , … Wilman, A. H. (2015). Validation of quantitative susceptibility mapping with Perls' iron staining for subcortical gray matter. NeuroImage, 105, 486–492. 10.1016/j.neuroimage.2014.11.010 25462797

[hbm25706-bib-0065] Sun, Y. , Ge, X. , Han, X. , Cao, W. , Wang, Y. , Ding, W. , … Xu, J. (2017). Characterizing brain iron deposition in patients with subcortical vascular mild cognitive impairment using quantitative susceptibility mapping: A potential biomarker. Frontiers in Aging Neuroscience, 9, 81. 10.3389/fnagi.2017.00081 28424610 PMC5371674

[hbm25706-bib-0066] Tao, Y. , Wang, Y. , Rogers, J. T. , & Wang, F. (2014). Perturbed iron distribution in Alzheimer's disease serum, cerebrospinal fluid, and selected brain regions: A systematic review and meta‐analysis. Journal of Alzheimer's Disease, 42, 679–690. 10.3233/JAD-140396 24916541

[hbm25706-bib-0067] Todorich, B. , Pasquini, J. M. , Garcia, C. I. , Paez, P. M. , & Connor, J. R. (2009). Oligodendrocytes and myelination: The role of iron. Glia, 57, 467–478. 10.1002/glia.20784 18837051

[hbm25706-bib-0068] Uchida, Y. , Kan, H. , Sakurai, K. , Arai, N. , Kato, D. , Kawashima, S. , … Matsukawa, N. (2019). Voxel‐based quantitative susceptibility mapping in Parkinson's disease with mild cognitive impairment. Movement Disorders, 34, 1164–1173. 10.1002/mds.27717 31091347

[hbm25706-bib-0069] Van Bergen, J. M. , Li, X. , Quevenco, F.‐C. , Gietl, A. , Treyer, V. , Meyer, R. , … van Zijl, P. C. (2018). Simultaneous quantitative susceptibility mapping and flutemetamol‐PET suggests local correlation of iron and β‐amyloid as an indicator of cognitive performance at high age. NeuroImage, 174, 308–316. 10.1016/j.neuroimage.2018.03.021 29548847 PMC5949258

[hbm25706-bib-0070] Wechsler, D. (1981). WAIS‐R manual: Wechsler adult intelligence scale‐revised. San Antonio, TX: Psychological Corporation.

[hbm25706-bib-0071] Wechsler, D. (1997). Wechsler adult intelligence scale‐III. San Antonio, TX: The Psychological Corporation.

[hbm25706-bib-0072] Whitwell, J. L. (2009). Voxel‐based morphometry: An automated technique for assessing structural changes in the brain. Journal of Neuroscience, 29, 9661–9664. 10.1523/JNEUROSCI.2160-09.2009 19657018 PMC6666603

[hbm25706-bib-0073] Zachariou, V. , Bauer, C. E. , Seago, E. R. , Raslau, F. D. , Powell, D. K. , & Gold, B. T. (2020). Cortical iron disrupts functional connectivity networks supporting working memory performance in older adults. NeuroImage, 223, 117309. 10.1016/j.neuroimage.2020.117309 32861788 PMC7821351

[hbm25706-bib-0074] Zecca, L. , Youdim, M. B. , Riederer, P. , Connor, J. R. , & Crichton, R. R. (2004). Iron, brain ageing and neurodegenerative disorders. Nature Reviews: Neuroscience, 5, 863–873. 10.1038/nrn1537 15496864

[hbm25706-bib-0075] Zhang, Y. , Wei, H. , Cronin, M. J. , He, N. , Yan, F. , & Liu, C. (2018). Longitudinal atlas for normative human brain development and aging over the lifespan using quantitative susceptibility mapping. NeuroImage, 171, 176–189. 10.1016/j.neuroimage.2018.01.008 29325780 PMC5857468

[hbm25706-bib-0076] Zheng, W. , Nichol, H. , Liu, S. , Cheng, Y.‐C. N. , & Haacke, E. M. (2013). Measuring iron in the brain using quantitative susceptibility mapping and X‐ray fluorescence imaging. NeuroImage, 78, 68–74. 10.1016/j.neuroimage.2013.04.022 23591072 PMC3843006

