# Supplementary figures and images for: Cortical iron mediates age‐related decline in fluid cognition
Source: Hum Brain Mapp. 2021 Dec 2;43(3):1047–60. doi: 10.1002/hbm.25706 (PMC8764476; doi:10.1002/hbm.25706)

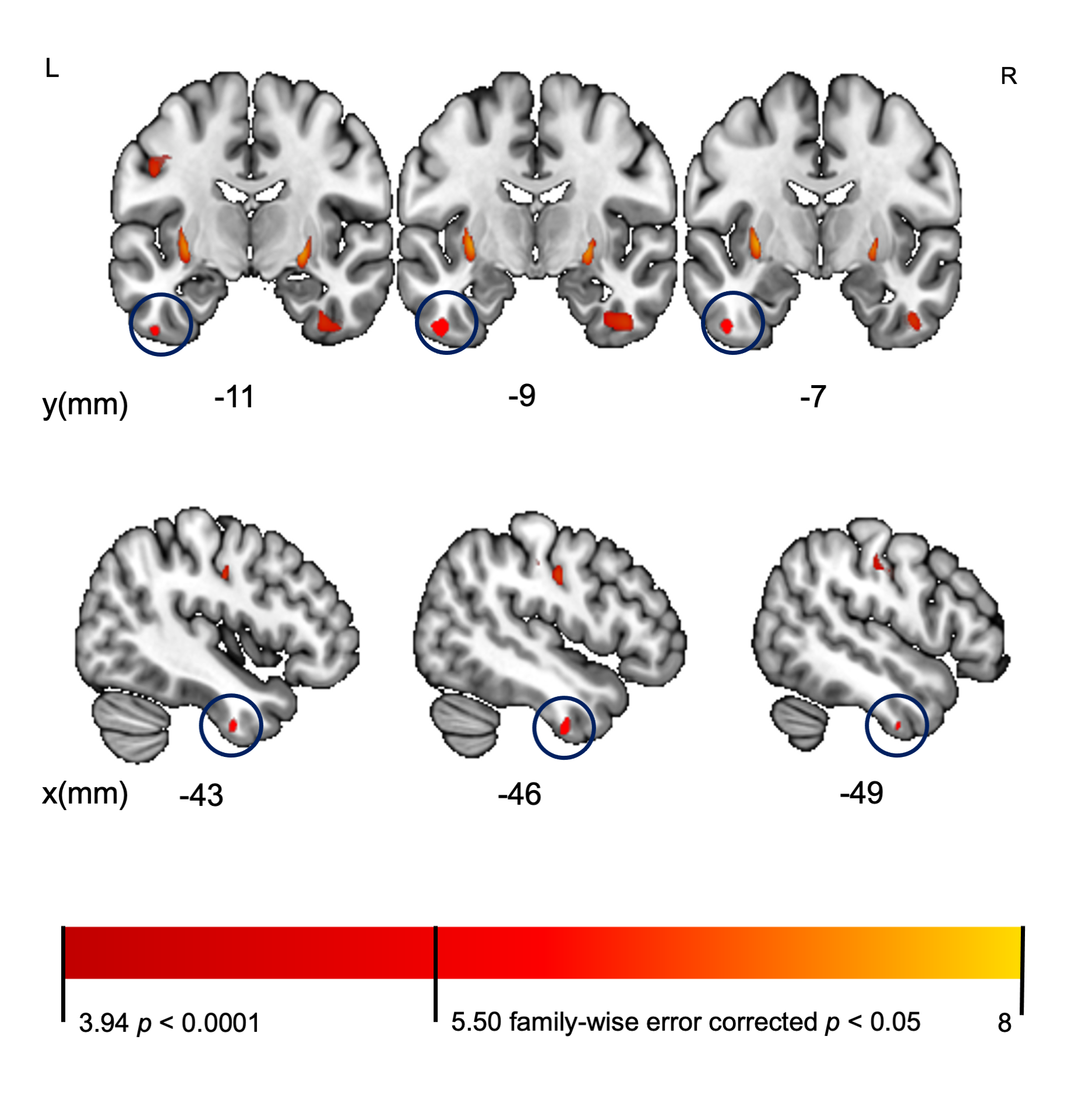

Supplement: Supplementary file 1 — Figure S1 Voxels correlated with fluid cognition including the left inferior temporal cortex. t‐values for voxels showing a negative fluid cognition and susceptibility association. Blue circles highlight the left inferior temporal cortex [file HBM-43-1047-s002.tiff]
