# Supplementary material for: Cortical iron mediates age‐related decline in fluid cognition
Source: Hum Brain Mapp. 2021 Dec 2;43(3):1047–60. doi: 10.1002/hbm.25706 (PMC8764476; doi:10.1002/hbm.25706)
Supplement: Supplementary file 2 — Table S1 Mediation of age and fluid cognition relation by average cluster susceptibility in the right inferior temporal cortex (RITC) controlling for gray matter volume [file HBM-43-1047-s001.docx]

Supplementary Materials

Supplementary Table 1. Mediation of age and fluid cognition relation by average cluster susceptibility in the right inferior temporal cortex (RITC) controlling for gray matter volume.

|  | Variable | Coeff | SE | t | p | Lower CI | Upper CI |
| --- | --- | --- | --- | --- | --- | --- | --- |
|  | RITC gray matter volume | 0.0239 | 0.0355 | 0.6738 | 0.5029 | -0.0470 | 0.0949 |
| A path | Age | 0.0004 | 0.0001 | 4.4360 | <0.0001 | 0.0002 | 0.0006 |
| B path | Fluid Cognition | -9.8771 | 4.6936 | -2.1044 | 0.0393 | -19.2565 | -0.4976 |
| A x B | Interaction | -0.0038 | 0.0018 | — | — | -0.0078 | -0.0006 |
| C’ path | Direct Effect of Age | -0.0403 | 0.0037 | -10.7951 | <0.0001 | -0.0478 | -0.0329 |
